# Agricultural and non-agricultural directions of bio-based sewage sludge valorization by chemical conditioning

**DOI:** 10.1007/s11356-021-15293-4

**Published:** 2021-07-19

**Authors:** Grzegorz Izydorczyk, Katarzyna Mikula, Dawid Skrzypczak, Krzystof Trzaska, Konstantinos Moustakas, Anna Witek-Krowiak, Katarzyna Chojnacka

**Affiliations:** 1 Department of Advanced Material Technologies, Faculty of Chemistry, Wrocław University of Science and Technology, Smoluchowskiego 25, 50-372 Wrocław, Poland; 2 School of Chemical Engineering, National Technical University of Athens, 9 Iroon Polytechniou Str., Zographou Campus, GR-15780 Athens, Greece

**Keywords:** Protein recovery, Acid hydrolysis, Alkaline hydrolysis, Thermal processing, Chemical conditioning, Fertilizer nutrients

## Abstract

This literature review outlines the most important—agricultural and non-agricultural—types of sewage sludge management. The potential of waste sludge protein hydrolysates obtained by chemical sludge conditioning was reported. The discussed areas include acidic and alkaline hydrolysis, lime conditioning, polyelectrolyte dewatering and other supporting techniques such as ultrasounds, microwave or thermal methods. The legislative aspects related to the indication of the development method and admission to various applications based on specified criteria were discussed. Particular attention was devoted to the legally regulated content of toxic elements: cadmium, lead, nickel, mercury, chromium and microelements that may be toxic: copper and zinc. Various methods of extracting valuable proteins from sewage sludge have been proposed: chemical, physical and enzymatic. While developing the process concept, you need to consider extraction efficiency (time, temperature, humidity, pH), drainage efficiency of post-extraction residues and directions of their management. The final process optimization is crucial. Despite the development of assumptions for various technologies, excess sewage sludge remains a big problem for sewage treatment plants. The high costs of enzymatic hydrolysis, thermal hydrolysis and ultrasonic methods and the need for a neutralizing agent in acid solubilization limit the rapid implementation of these processes in industrial practice.

**Graphical abstract:**

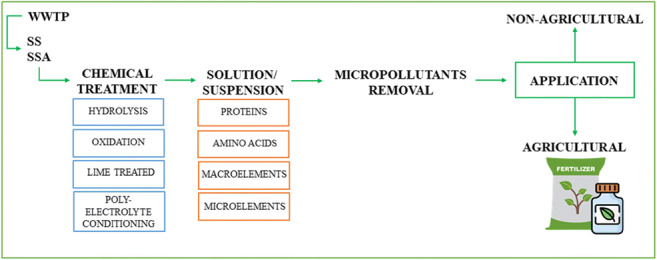

## Introduction

Wastewater treatment plants generate huge amounts of primary (from the first stage of wastewater treatment), secondary (from the biological treatment stage) and tertiary sludge (from an additional stage of biogenic removal), which require utilization. It is estimated that this particular stage (third stage) is the most cost-intensive and may take up to 60% of the total operating costs (Pilli et al. 2015) and can generate up to 40% of total greenhouse gas emissions (Anjum et al. 2016). An increasing number of stringent regulations controlling the directions of sludge management force the search for effective techniques that are least detrimental to the environment. The reduction of sludge production, its microbiological stabilization and recovery of valuable components and energy (Khanal et al. 2007) can be carried out through aerobic and anaerobic digestion (Anjum et al. 2016), wet oxidation, hydrothermal processes, pyrolysis and gasification, all of which that are fully described in the reviewed article (Tyagi and Lo 2013a) are used. Processed sludge, initially pre-treated (stable biosolids), can be recycled or landfilled. The most popular directions for reuse are soil application, composting and incineration (Paz-Ferreiro et al. 2018). Interesting option of use may also be the construction industry (Ahmad et al. 2016), production of bioplastic (Tyagi and Lo 2013a), combustible materials, and the recovery of volatile fatty acids (Cies̈lik et al. 2015; Fang et al. 2020), as evidenced by numerous literature reviews.

Sewage sludge is a heterogeneous material, being a mixture of microorganisms, non-biodegradable organic substances, consisting mainly of water and a small number of solids (about 10%). This type of material is problematic in processing (Pilli et al. 2015). The cell wall is made up of hard-to-degrade biopolymers such as polysaccharides (lignin, cellulose, hemicellulose) and peptidoglycan. Therefore, preliminary disintegration of cells is essential to allow the components to leak from inside the cells into the aqueous phase (Khanal et al. 2007). Sludge pretreatment includes thermal, mechanical, chemical or biological methods (Carrère et al. 2010; Zhou et al. 2007) that are targeted to reduce the amount of sludge, increase the efficiency of energy production and release nitrogen and phosphorus fractions from inside the cells while increasing the availability of intracellular organic compounds.

Thermal pretreatment (TPT) includes high (usually between 150 and 250 °C) and low temperature (60–100 °C) thermal hydrolysis, which means increased sludge temperature, usually induced by hot steam, accelerates cell decomposition, and increases biogas production and improves sludge dehydration (Pilli et al. 2015). An extensive literature review of hydrothermal technologies, including a discussion of the recovery of valuable components from sewage sludge using thermal hydrolysis, subcritical wet oxidation and supercritical wet oxidation, was also presented by Suárez-Iglesias et al. (Suárez-Iglesias et al. 2017). Another thermal method—referred to as an environmentally friendly technique, mainly due to its reduced emissivity—that can support sludge treatment is microwave radiation, described in detail by Tyagi and Lo (2013b). Bio-drying, which uses the heat generated by the aerobic activity of microorganisms, aided by the airflow, is considered an effective technique for the pretreatment of sludge for agricultural purposes (Pilnáček et al. 2019). Khanal et al. (2007) presented an excellent literature review on the use of ultrasonic treatment for sewage sludge disintegration. This method—expensive though it is—in addition to facilitating cell degradation also results in the decomposition of hard-to-degrade organic compounds due to the formation of free radicals. Due to its high protein content and global mass production (approximately 11 million Mg of d.m. in the EU (Madrid et al. 2020)), sewage sludge can be a source of plant biostimulators (including peptides or free amino acids). The increased bioavailability of these compounds can only be achieved by composting or hydrolysis (Xu and Geelen 2018). A review on sewage sludge management has already been published by Cies̈lik et al. (2015), but in their paper, new solutions using as the example sewage sludge management in Wastewater Treatment Plant “Wschód” in Gdańsk were described. The focus was mainly on thermal methods (drying, conventional incineration, co-incineration) and sludge stabilization by different methods (using earthworms or drying bed, anaerobic stabilization) for waste management in agriculture or soil reclamation (Cies̈lik et al. 2015).

This literature review includes a discussion of the most important directions of sewage sludge management, both agricultural and non-agricultural, with particular emphasis on the potential of hydrolysates obtained from chemical sludge treatment. Valuable data have been collected to characterize the problem of sludge generation and its different compositions, and current management methods (agriculture, composting, landfilling) have been identified. Legal aspects on which the direction of sewage sludge management depends were also discussed. Particular emphasis was placed on the potential of hydrolysates obtained from the chemical treatment of sewage sludge, which is a novelty of this work. The discussed areas include in particular acidic and alkaline hydrolysis, lime conditioning, polyelectrolyte dewatering and other supporting methods such as ultrasound, microwave or thermal methods processes. To the best of our knowledge, there is no overview available covering this topic.

## Characteristics of sewage sludge

Secondary sewage sludge generated by wastewater treatment plants is a by-product of biological wastewater treatment and originates from secondary settlers. A part of the sludge is recycled to the aeration chamber as recycled activated sludge (RAS) to improve the rate performance of wastewater treatment. *The other part, waste activated sludge (WAS), is usually transported with special pumps to fermentation chambers for biogas production.* The post-fermentation residue or the excess sludge requires utilization. Until now, no efficient and cost-effective method has been developed.

**Fig. 1:**
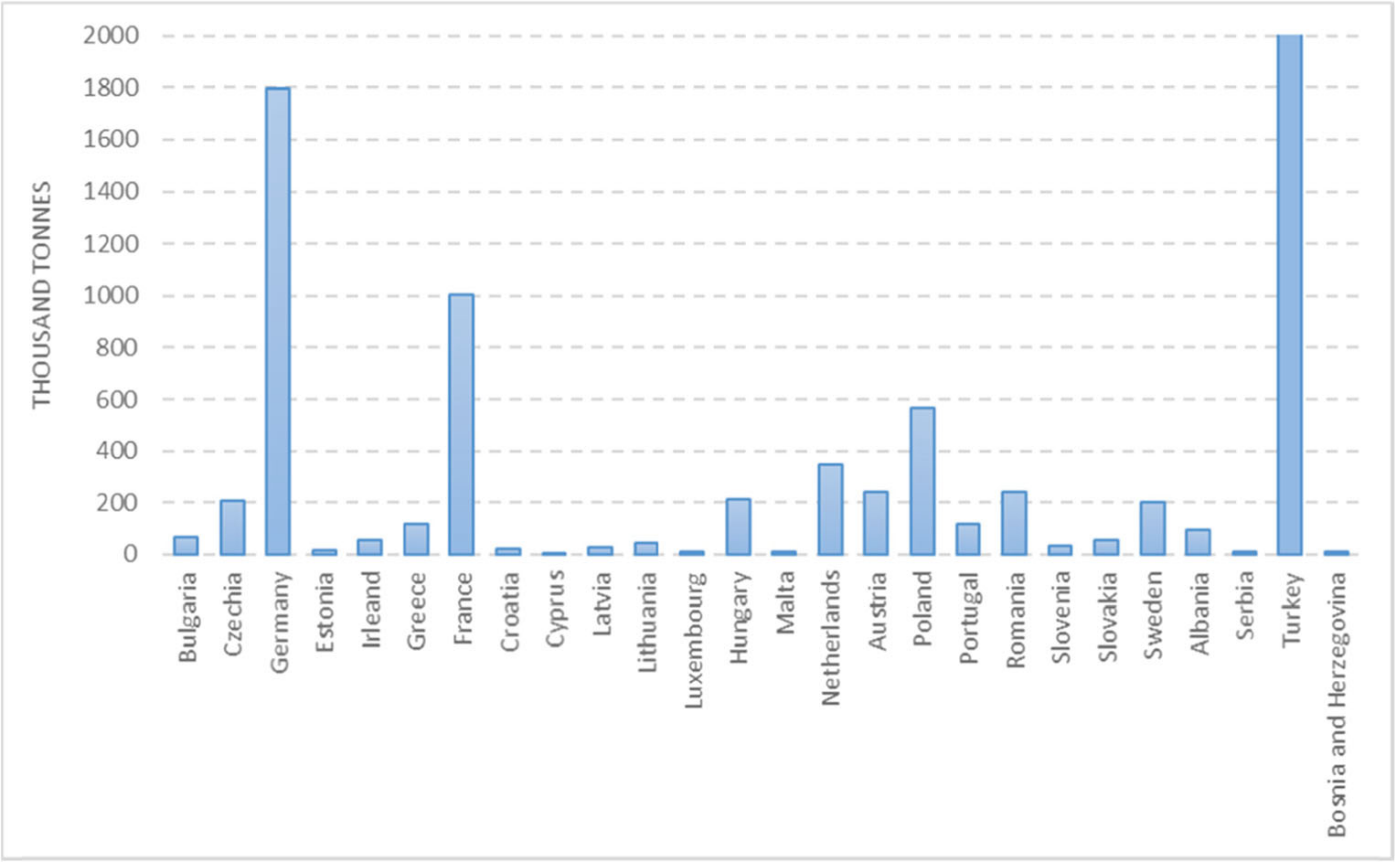
Production of sewage sludge (2016) (“Eurostat - Data Explorer,” 2020)

Sludge production is increasing annually due to population growth and intensive urbanization (Fig. 1). In 2017, 45 MT of dry excess sewage sludge mass was produced globally (Gao et al. 2020a, b).

Sludge has a semi-liquid consistency with high water content (up to 98%). Activated sludge is rich in organic matter, phosphorus and nitrogen, which are valuable components for fertilizers. Heavy metals and pathogens may also be present in sludge, which poses a risk to its further use as a resource. Therefore, it is necessary to apply appropriate treatment techniques to reduce volume (dewatering), remove the undesirable components and recover valuable materials.

**Fig. 2:**
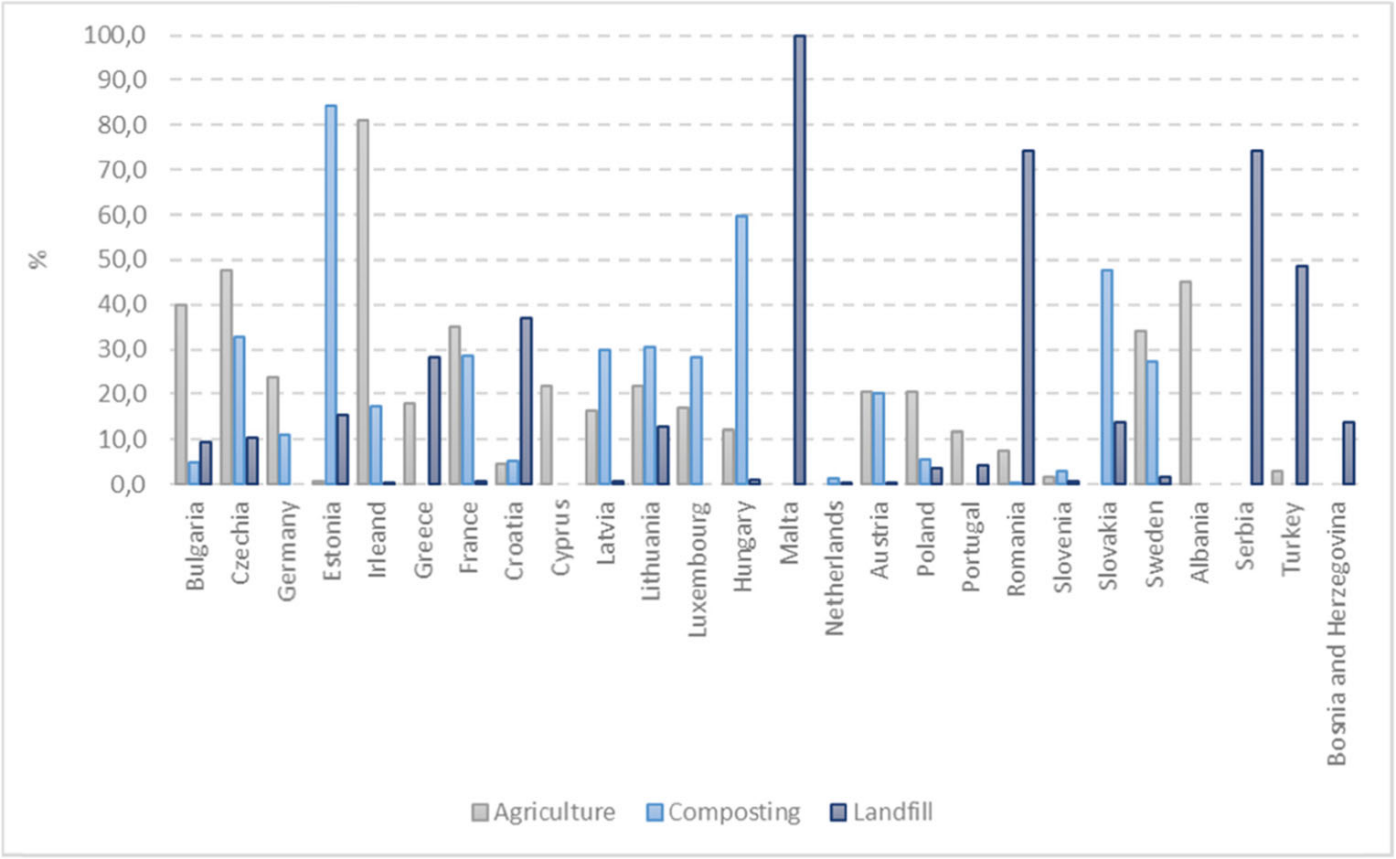
Sludge management (2016) (“Eurostat - Data Explorer,” 2020)

Restrictive regulations (Table 1) indicated the purpose of wastewater treatment (decreasing the discharge of pollutants into surface waters), limited the maximum allowable content of heavy metals (Table 2) in sewage sludge and recommended its use as the substrate for other processes (waste valorization for agricultural purposes or energy recovery) (Inglezakis et al. 2011). All these activities are consistent with the assumptions of the closed-loop economy; the main objective of which is to reduce the amount of waste and reuse it for material and energy recovery. The directions of sludge management in European countries are shown in Fig. 2.

**Table 1:** Legislative aspects of sewage sludge management in the EU

Directive	Sector	Targets
1986/278/EEC	Directive on the use of sludge in agriculture	Limits the heavy metal content of sewage sludge used in agriculture
1991/271/EEC	Directive on urban wastewater treatment	Protection of the environment from adverse effects of urban wastewater, promotion of sewage sludge reuse
1996/61/EC	Directive on pollution prevention and control	Integrated prevention and reduction of pollution
1999/31/EC	Landfill Directive	Reduction of the disposal of biodegradable waste in landfills
2000/60/EC	Water Framework Directive	Gradual reduction of discharges of pollutants from wastewater into the aquatic environment
Directive 2000/76/EC	Directive on the incineration of waste	Sets emission limit values for the incineration and co-incineration of waste
2008/98/EC	Waste Framework Directive	Regulates waste recycling and induces a reduction in waste generation
2009/28/EC	Directive on Renewable Energy	Use of sewage sludge to produce energy (i.e. biogas)

**Table 2:** Elemental composition of sewage sludge

Sludge type	N [%]	P [%]	K [%]	Ca [%]	Mg [%]	Heavy metals [mg/kg]										Reference
						Cu	Fe	Zn	Pb	Ni	Cr	Cd	Mn	As	Hg	
AD SS	4.19	2.00	1.16	8.34	1.06	917	n.a.	10.9	487	363	70.4	n.a.	n.a.	n.a.	n.a.	(Uysal et al. 2017)
Concentrated excess sludge	0.07–0.09	0.02-0.03	n.a.	n.a.	n.a.	n.a.										(Bi et al. 2014)
SS (d.m.)	0.06 N-NH_4_	0.51 P-PO_4_	n.a.	n.a.	n.a.	259	n.a.	1298	300	63	550	n.a.	n.a.	n.a.	n.a.	(Stylianou et al. 2007)
SS	8.34	2.59 P_2_O_5_	0.35 K_2_O	0.12 CaO	0.41 MgO	n.a.										(Santos et al. 2020)
Fermented sludge	6.6	1.6	n.a.	n.a.	n.a.	44.8	n.a.	72	13.4	7.3	9.4	1.12	n.a.	4.18	0.95	(Janas et al. 2018)
Dewatered sludge	6.7	1.8	n.a.	n.a.	n.a.	53	n.a.	865.2	16.6	8.8	11.3	1.35	n.a.	5.2	1.14	(Janas et al. 2018)
SS	1.6	1.3	0.8	n.a.	n.a.	186	232	161	28.5	54.7	44.3	32.2	260	n.a.	n.a.	(Latare et al. 2014)
Composted SS	2.5	3.5 P_2_O_5_	0.4 K_2_O	n.a.	n.a.	330	n.a.	340	9.4	n.a.	n.a.	1.8	n.a.	n.a.	n.a.	(Phung et al. 2020)
SS	112 mg/kg NH_4_-N, 32 mg/kg NO_3_-N	0.24	1.54%	n.a.	n.a.	90	11358	534	n.a.	n.a.	n.a.	n.a.	2839	n.a.	n.a.	(Koutroubas et al. 2020)

## Sludge treatment methods

**Fig. 3:**
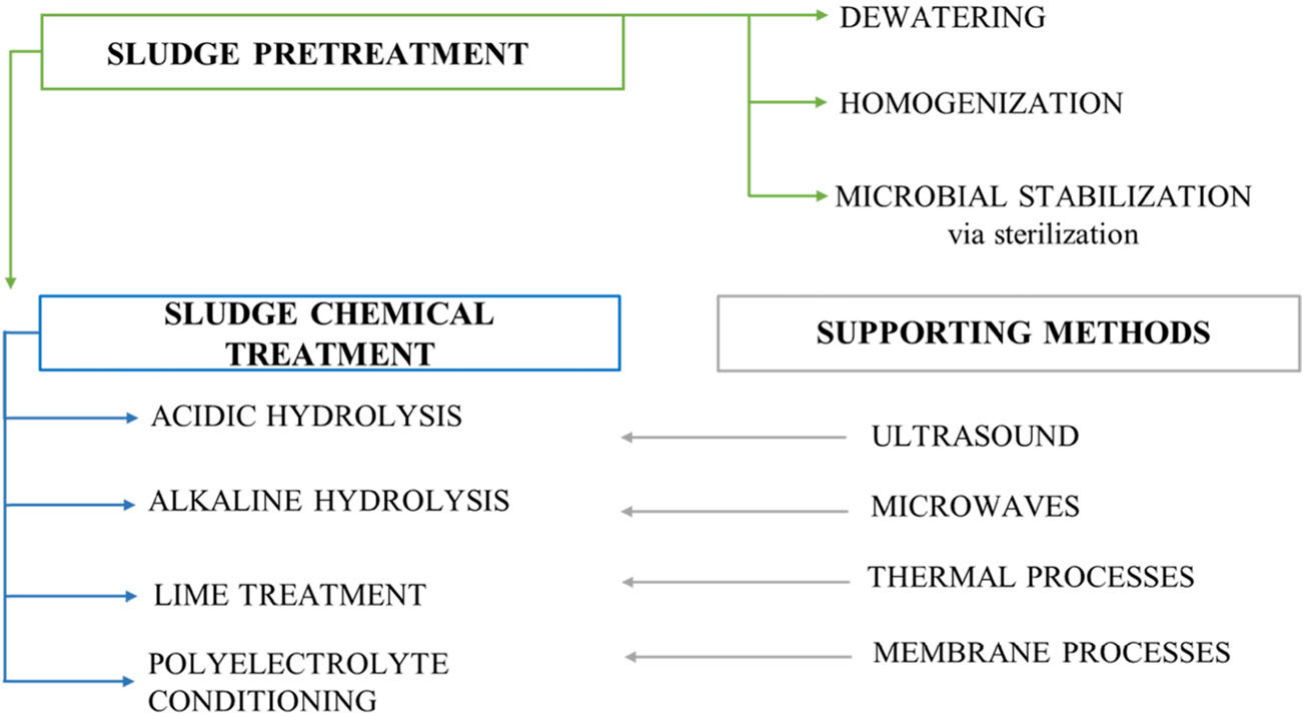
Sludge treatment

The first step in sludge valorization is pre-treatment (Fig. 3), what usually includes mechanical processes leading to dewatering and homogenization. In exceptional cases, sterilization is also used to ensure microbiological stability (Zhen et al. 2017).

Then, the treatment stage is carried out using various methods, of which chemical methods are the most commonly implemented. Conditioning with alkaline, acidic, oxidizing agents, as well as electrolysis or hydrodynamic cavitation (Cai et al. 2018), are more interesting as compared to anaerobic digestion or dewatering prevalent up to now/earlier. Chemical treatment requires simple equipment, has a quick rate and high efficiency.

### Hydrolysis

Alkaline hydrolysis decomposes flocs and cells thus accelerating solubilization (Cassini et al. 2006) and subsequent anaerobic decomposition (Kim et al. 2003). During treatment, water is released from inside the cells and flocs, which cannot be done by traditional methods (Neyens et al. 2003). The rapidity of the reaction causes denaturation of proteins and leads to the saponification of lipids and the hydrolysis of RNA, which results in the release of components from internal structures. The presence of OH^-^ ions also triggers/results in the ionization of carboxylic groups, swelling and consequently to faster solubilization (Neyens et al. 2004). The addition of an acid initiates the hydrolysis of proteins and polysaccharides and reduces electrostatic repulsion, which in turn causes the coagulation of sediment particles. Strong acids and hydroxides support sludge dewatering and affect its sanitization, which allows for its subsequent safe management (Bian et al. 2018; Li et al. 2008).

#### Acidic hydrolysis

Acidic hydrolysis of microbiological proteins is an effective method of obtaining amino acids. For this purpose, hydrochloric acid in liquid and gaseous forms is often used (Chen et al. 2007). Liu et al. (2009) presented the hydrolysis of sewage sludge from a sewage treatment plant in China using hydrochloric acid (2 M) at a temperature of 105°C. They achieved an 80% protein extraction within 24 h. The decolourization of active carbon purified the amino acid solution. It is because of HCl that hydrolysate contains chlorides. It limits its use in the nutrition of plants since fruits and vegetables are sensitive to chlorides.

Conditioning sewage sludge with inorganic and organic acids is often used to recover phosphorus, nitrogen, and other valuable elements (Cieślik and Konieczka 2017). Low pH favours leaching of macro- and microelements, but the presence of toxic metals often negatively affects the quality of hydrolysate (Güney et al. 2008). The effect of acid hydrolysis of the digested sludge on metal recovery was investigated. Inorganic acids (hydrochloric acid, sulphuric acid(VI), nitric acid(V)) and organic acids (acetic, citric and oxalic acid) were used in the experiment. Optimal process conditions were determined for hydrolysis with oxalic acid (0.5 M), where the sludge to acid ratio was 1:10 and hydrolysis time was 60 min. Donnan dialysis separated the components. The recovery of iron, magnesium, aluminium, sodium and potassium was obtained at 5, 39, 3, 58, 62 and 68%, respectively. Hydrolysis with oxalic acid reduced the release of calcium, which negatively affects the purity of struvite, and the use of dialysis eliminated undesirable metal ions (Uysal et al. 2017). Chen et al. (2020) proposed the hydrolysis of sludge from the municipal sewage treatment plant with oxalic acid. For performance comparison, the sludge was also treated with sulphuric and hydrochloric acid. It was found that conditioning with oxalic acid causes hydrolysis of polysaccharides, especially pectins, and works as a chelating agent for selected metal species. The process brought high values of soluble forms of ferrous, magnesium, aluminium and calcium ions, which may play the role of flocculant at later stages. In the case of protein, the hydrolysis yield decreased about twice, as compared to the process conducted with sulphuric and hydrochloric acid.

#### Alkaline hydrolysis

Since alkaline hydrolysis does not require special equipment, it significantly reduces its costs. Alkaline media can effectively disintegrate cells and flocs, releasing intracellular organic compounds and water, promoting sludge dewatering (Li et al. 2008). Sewage sludge with alkaline pH media positively influences protein breakdown into short peptides and amino acids (Kim et al. 2003). The effect of pH on the hydrolysis of sewage sludge from a municipal sewage treatment plant in China on the content of soluble protein, volatile fatty acids and carbohydrates in hydrolysate was studied. The experiment was conducted within the pH range from 4 to 11, using 2 M sodium hydroxide (NaOH) and 2 M hydrochloric acid (HCl). Conducting the process under alkaline conditions increased the content of soluble proteins and carbohydrates in hydrolysate as well as that of fatty acids. Hydrolysis also released soluble phosphorus and ammonia. Alkaline hydrolysis made it possible to recover these components by precipitation and dewaxing. During hydrolysis, methane production increased with decreasing pH (Chen et al., 2007). Otherwise, sodium hydroxide (0.1 M) has been shown to increase biogas production by about 30% and enhance the rate of organic matter degradation by about 40% compared to controls. The results showed that the proportion of soluble forms of volatile fatty acids as well as polysaccharides increased significantly (Li et al. 2013). Anaerobic digestion with alkalization was also used to recover phosphorus from sewage sludge. The hydrolysis of sewage sludge from sewage treatment plants in China was carried out in the presence of sodium hydroxide (pH 9–13) with continuous mixing. The highest effects of hydrolysis were achieved at pH 10. It was found that rising pH increased phosphorus recovery (preferably pH 13) (Zhou et al. 2020). The most commonly used material for the recovery of phosphorus is in the form of hydroxyapatite. Phosphorus is precipitated in the form of, e.g., struvite (Kim et al. 2015), which is closely related to the presence of metals such as potassium, sodium or calcium (Pastor et al. 2008). Pastor et al. (2008), in their work, presented the possibility of selective recovery of P and N from activated sludge through its thermal (50–80°C) and alkaline treatment with sodium hydroxide (0.001–1 M). Phosphorus was recovered at the level of 60%, and nitrogen at 90%. It was found that the recovery of elements increases proportionally to hydroxide concentrations, while the influence of temperature is only significant in the case of low concentrations of hydroxides.

Soluble forms of proteins and polysaccharides can also be obtained by extraction with alkali. Hydroxides ionize the groups present in polysaccharides and proteins, which leads to the mutual clogging of components, thus increasing their solubility. This method is not often applied because of the problems related to polymer analysis (McSwain et al. 2005). García Becerra et al. (2010) extracted proteins, polysaccharides and lipids from sewage sludge with sodium hydroxide (50% m/m). Also, in this case, better effects were observed at high pH (12.8) (extracts up to 75%).

*An important variable in alkaline hydrolysis is the ratio of waste material to hydrolysis medium*. The effect of sodium hydroxide and calcium hydroxide doses on the dewatering capacity and hydrolysis of sludge from sewage treatment plants were analyzed within the concentration range of 0 to 0.5 mol/L, at temperatures from 0 to 40°C. The dose of 0.5 mol/L of sodium hydroxide promised to be the most effective for decomposition. After 30 min of solubilization, the efficiency was ca. 70%. With the increasing dose, higher efficiency was observed. Calcium hydroxide has a significant effect on sludge dehydration. This is caused by the flocculation of the fragmented flocs and the dissolution of suspended solids by calcium cations (Li et al. 2008). Calcium hydroxide reduces most indicatory microorganisms from the *cola* group and pathogens such as *Salmonella* and *Dechloromonas*. The problem is among other caused by some antibiotics (e.g. fluoroquinolones, cotrimoxazole, aminoglycosides), which are characterized with the highest activity in alkaline environments (Lopes et al. 2020).

In several cases, a multi-stage treatment is used to increase the efficiency of sludge solubilization. Siami et al. (2020) present a three-stage process consisting of thermal treatment (75–90°C), alkaline treatment (sodium hydroxide (pH 12)) and oxidative conditions (hydrogen peroxide). The last one is used for anaerobic biomass decomposition. In this case, the degradation of proteins, cells or phospholipids is initiated by the formation of free radicals (NO, HO) and may affect methane production (Feki et al. 2015). The use of this type of sludge treatment significantly increases the solubility of organic fractions. The study also controlled the production of methane, which increased by about 90% compared to the control sample (Siami et al. 2020) (Table 3).

**Table 3:** Conditions for the process of sewage sludge hydrolysis

Hydrolysis type	Reagents	Reagent concentration	L:S ratio	Time	Before hydrolysis	After hydrolysis	Temperature (°C)	Reference
Acidic	H_2_SO_4_ HCl	n.a.	n.a.	40 min	Bound water 3.32 (%) Protein 0.16 Polysaccharide 0.61 K 1.6 Na 0.6 Fe 0.01 Mg 0.4 Al 0.1 Ca 0.2 (mg/g)	Bound water 2.84 (%) Protein 3.18 Polysaccharide 0.87 K 2.7 Na 0.8 Fe 6.6 Mg 2.7 Al 1.2 Ca 8.0 (mg/g)	n.a.	(Chen et al. 2020)
Acidic	HCl	2M	10:1	24h	Cu 187.97 Zn 612.50 Fe 568.48 Mn 543.98 Ni 37.93 Pb 61.08 Cd 3.16 Cr 26.33 Hg 3.87 As 15.85 (mg/kg)	Cu 355.89 Zn 1073.33 Fe 689.76 Mn 323.28 Ni 36.41 Pb 66.43 Cd 1.90 Cr 42.28 Hg 3.26 As 14.28 (mg/kg)	105°C	(Liu et al. 2009)
Acidic	H_2_SO_4_	0.3–0.7 M	5–15:1 (mL/g)	30–90 min	Ca 83380 Mg 10620 K 11560 Na 11280 Al 6831 Fe 6140 Zn 10960 Cu 9171 Cr 70.42 Pb 487.4 Ni 363.3 (mg/kg)	Ca 82710 Mg 9902 K 10967 Na 10710 Al 6534 Fe 5730 Zn 10855 Cu n.a. Cr n.a. Pb n.a. Ni n.a. (mg/kg)	Room temperature	(Uysal et al. 2016)
Alkaline Acidic	NaOH HCl	2 M 2 M	n.a.	8 day	Protein 8180 Carbohydrate 1522 Lipid and oil 131 (mg/L)	Protein 112.25 Carbohydrate 30.11 Lipid and oil n.a. (mg/L)	Room temperature	(Chen et al. 2007)
Alkaline	NaOH	10 M	n.a.	0.5–12h	Total phosphorus (TP) 200, total nitrogen (TN) 490, PO43—P125, NH^4+^-N 50 (mg/L)	Total phosphorus (TP) 205, total nitrogen (TN) 525, PO43—P140, NH^4+^-N 80 (mg/L)	Room temperature	(Bi et al. 2014)
Alkaline	NaOH	0.005–0.5 M	n.a.	30 min	Soluble chemical oxygen demand 3052 (SCOD, mg/L)	Soluble chemical oxygen demand 6000, 1800 (SCOD, mg/L)	Room temperature	(Li et al. 2012)
Alkaline	NaOH	50%	n.a.	48 h	Protein yield 17 (g/100g), carbohydrate yield 6 (g/100g), lipid yield 0.7 (g/100g)	Protein yield 20 (g/100g), carbohydrate yield 7 (g/100g), lipid yield 1.0 (g/100g)	Room temperature	(García Becerra et al. 2010)
Alkaline	NaOH Ca(OH)_2_	0.05–1 M NaOH 0.02–0.5 M Ca(OH)_2_	n.a.	24h	Soluble chemical oxygen demand 275 (SCOD, mg/L)	Soluble chemical oxygen demand 3100–4800 (SCOD, mg/L)	0–40°C	(Li et al. 2008)
Alkaline	NaOH KOH Ca(OH)_2_ Mg(OH)_2_	n.a.	n.a.	n.a.	Soluble chemical oxygen demand 2250 (SCOD, mg/L)	Soluble chemical oxygen demand 7500 (SCOD, mg/L)	Room temperature	(Kim et al. 2003)
Alkaline	NaOH	0.001–1 M	n.a.	n.a.	n.a.	K 96.0 P 562.1 Mg 155.3 Ca 119.7 (mg/L)	50–80°C	(Pastor et al. 2008)
Alkaline	NaOH	5% m/m	n.a.	n.a.	n.a.	n.a.	Room temperature	(Zhou et al. 2020)
Alkaline Thermal	Sodium bicarbonate	n.a.	n.a.	1–10 h	Chemical oxygen demand COD 0 (mg/L)	COD 69000 (mg/L)	50–90°C	(Vlyssides and Karlis 2004)
Alkaline Thermal	NaOH	5 M	n.a.	15 min	Soluble chemical oxygen demand CODs 1.2 (g/L)	CODs 7.6 (g/L)	120°C	(Vigueras-Carmona et al. 2011)
Alkaline Oxidation	NaOH H_2_O_2_	n.a.	n.a.	24h	CODs 4.81(g/L) Protein 3.35 (g/L) Polysaccharide 0.46 (g/L)	CODs 19.29 g/L Protein 3.55 (g/L) Polysaccharide 0.42 (g/L)	75–90°C	(Siami et al. 2020)
Alkaline Oxidation	NaOH H_2_O_2_	5 M 30% m/m	n.a.	2, 4, 6, 8, 24, 48 and 72h	COD solubilization 10.5%	COD solubilization 28%	28°C	(Feki et al. 2015)

### Lime-treated sewage sludge

Since the purpose of sewage sludge treatment is to obtain a useful secondary raw material and reduce environmental damage, an assessment of the hygienic and sanitary properties of sludge is necessary (Zhuravlev et al. 2019). To this end, lime is commonly used. The addition of an alkaline stabilizer increases the temperature and pH of sludge, which leads to the elimination of pathogenic organisms and reduces the problem of odour (Wong and Fang 2000). Santos et al. (2020) conducted sanitary tests after chemical treatment to determine the presence of microorganisms in sewage sludge from 12 municipal sewage treatment plants. Alkaline industrial waste (limestone mud, volatile coal ash, eggshells) and calcium hydroxide were used as chemical conditioning agents. Each of these materials was mixed with the precipitate and contacted for 24 h. Among the materials tested, only eggshells (a source of CaO obtained during calcination) and calcium hydroxide eliminated *E. coli* (for any dose tested: 0.05–0.15g/g SS). Studies show the need to disinfect sewage sludge before it is used, especially in agriculture (Santos et al. 2020). Experimental alkaline treatment to eliminate pathogens (bacteria and worm eggs) was also carried out by Lopes et al. (2020). Sewage sludge was mixed with hydrated lime. It was noted that some bacteria (*Geobacter* and *Geothrix*) were still present in the lime-treated sewage sludge despite strongly alkaline conditions (pH >12). Antibiotic resistance genes were detected. Their presence in samples is currently not regulated by sanitary regulations, but their monitoring is important for human health (Lopes et al. 2020).

An important environmental issue is the content of toxic metals in sewage sludge, limiting their potential use in the agricultural and non-agricultural sectors. A significant amount of metals (80–90%) from sewage accumulates in sewage sludge (Janas et al. 2018). Healy et al. (2016) analyzed lime-treated sewage sludge. X-ray fluorescence revealed that the concentrations of all the metals were within limits prescribed by European Union regulations. Extensive analysis showed high levels of toxic elements (Se, Sr, Sn) that are not covered by these regulations (Healy et al. 2016). The origin of wastewater affects the content of toxic elements and their form (dissolved, precipitated or co-precipitated with oxides). Janas et al. (2018) studied the effect of calcium oxide on the mobility of toxic metals (Cu, Zn Ni, Cd, Hg) from sewage sludge (fermented and dehydrated). The dissolved oxygen content or changes in pH may cause the release of various elements from the sludge. Based on the results, copper was the most mobile and bioavailable metal ion. Other metal ions showed a lower leaching efficiency from sewage sludge (the smallest for Ni and Cd). Different doses of alkaline agents boosting composting (1.5–75g/10g d.m. sludge) did not significantly affect the degree of leaching of toxic elements (Janas et al. 2018). However, lime (2000–8000 mg/L) combined with aeration can remove metals from leachate. Cu, Pb, Fe, Mn, Zn, Ni and Cr concentrations decreased by 59, 58, 67, 76, 55, 29 and 64%, respectively (Cecen and Gursoy 2000).

### Polyelectrolyte conditioning of sewage sludge

Sewage sludge dewatering is considered an expensive treatment and its effectiveness is unsatisfactory, especially in cases where SS is characterized by a high content of organic matter or microorganisms, clogging the filters. Polyelectrolytes facilitate the SS dehydration process. Due to the neutralization of charge and intermolecular bridging, fine particles combine into larger aggregates and are easier to separate on filters. The efficiency of wastewater conditioning depends on their molecular weight (MW) and their charge density (CD). The larger the MW, the larger the flocs are and the more resistant to hydrodynamic shear (Thapa et al. 2009). Polyelectrolytes with a charge density within the range of 0.18–1.42 mequiv/g are responsible for forming the strongest flocs (Gray and Ritchie 2006). Compared to inorganic flocculants (ferrous chloride, lime), charged polymers are biodegradable and can be used at smaller doses (Saveyn et al. 2005). Traditionally, cationic, anionic or nonionic polymers are used to condition sludge. However, the drainage efficiency of SS can be increased by using a mixture of cationic and nonionic polyelectrolyte (Lee and Liu 2000).

### Methods enhancing chemical sewage sludge treatment

#### Ultrasound-assisted methods

The use of ultrasounds in sludge treatment is known in literature and accurately described. The purpose of sonication is to disintegrate sludge and the microorganisms in it, such as bacteria, fungi and viruses. This has a hygienic significance, which furtherly allows for wider use of sludge. As a result of disintegration induced by ultrasounds, chemical oxygen demand (COD) and the content of chemical compounds such as proteins, amino acids and nucleic acids increase (Zhang et al. 2007). Tian et al. (2015) described the pretreatment of sewage sludge with ultrasound-assisted alkaline hydrolysis by means of which they achieved a significant increase in COD (from 1200 to 11,000 mg/L). Through fluorescence spectroscopy, they detected substances originating from the decomposition of microorganisms, thus confirming the hygienization efficiency of ultrasounds. It was also found that the use of sonification favoured the biodegradation of sludge. Pretreatment leads to an increase in the content of humic substances as a product of the decomposition of organic molecules of organic matter with high molecular weights. Farooq et al. (2009) also indicate an increase in sludge biodegradability due to ultrasound treatment. This is caused by increased efficiency of processes such as solubilization and anaerobic digestion, which in turn is due to microbial cell disintegration, flocculation and decomposition of bio-solids as well as reduction of sludge in mass and volume. It was also found that the use of ultrasounds to support hydrolysis ensures greater amino acid extraction (Gao et al. 2020a). Sludge decomposition boosted by ultrasounds has certain requirements and limitations. It is beneficial if the sludge to be treated has high water content: the higher the water content, the more efficient the process is. The degree of sludge decomposition varies depending on the time of sonification and the density of ultrasonic wave power and energy. This relationship is directly proportional (Zhang et al. 2007).

#### Microwave-assisted methods

Microwaves can also support the pretreatment of sludge. Technological solutions based on microwaves bring similar results as the application of ultrasounds. First of all, the decomposition of flocs and disintegration of walls and cell membranes of microorganisms is achieved, yielding higher biodegradation of sludge, increased susceptibility to sludge fermentation and their hygienization by neutralizing pathogens (Grübel et al. 2019). Most often, microwaves support digestion in acid or alkaline hydrolysis, as evidenced in the literature. Wang et al. (2017) describe the treatment of sewage sludge with sodium hydroxide and microwaves to increase the recovery of struvite phosphorus. In this study, the application of microwaves is primarily aimed at the decomposition of organic compounds containing phosphorus and transforming it into an inorganic form that can be subjected to precipitation in struvite crystals. Thanks to the increased efficiency and hygienization, the process is cheaper and the obtained struvite is suitable for agricultural purposes. Another study describes the use of microwaves as support for acid and alkaline hydrolysis of dairy sludge. Although in both cases, an increase in hydrolysis efficiency in the presence of microwaves is achieved, it was noted that alkaline hydrolysis in the presence of microwaves is much more efficient than acid hydrolysis supported by microwaves. The yield increase in alkaline hydrolysis was 30% higher than in acid hydrolysis. This leads to a higher yield of biogas (Beszédes et al., 2009). Literature data show that the use of microwave methods has positive results also in dry methods, such as pyrolysis (Zhang et al. 2018) as well as non-hydrolyzing wet torrefaction (Zheng et al. 2020).

#### Thermal hydrolysis

Thermal hydrolysis is also a well-known way of sludge treatment. As with other methods, hydrolysis at elevated temperatures shortens the chains of macromolecular organic compounds and hygienizes the material. Thanks to this solution, the sludge is more susceptible to other forms of treatment as its rheological properties change (Barber 2016). Literature data recommends using temperatures within the range of 140–170 °C, 5–35min contact time and single sudden decompression (Sapkaite et al. 2017). Thermally assisted hydrolysis serves as a pretreatment before sludge fermentation to obtain methane. This process leads to the dehydration of sludge, which increases the fermentation efficiency (Xu et al. 2019). Methane as a fuel has a reduced carbon footprint. Environmental considerations are also relevant for other applications of post-thermal sludge hydrolysis. Literature reports that similarly to other supporting methods, temperature also increases the biodegradability of sludge. Increased temperature also influences the level of micropollutants, such as drugs, hormones, which, as organic compounds, are quite easily thermally degraded (Taboada-Santos et al. 2019a). Unit processes related to thermal hydrolysis and the equipment used for this purpose influence process efficiency. From an economic point of view, it is energy and waste disposal costs that are important. The energy costs associated with achieving high temperatures are offset by savings resulting from no waste landfilling fees (Taboada-Santos et al. 2019b).

## Problem of micropollutants

**Fig. 4:**
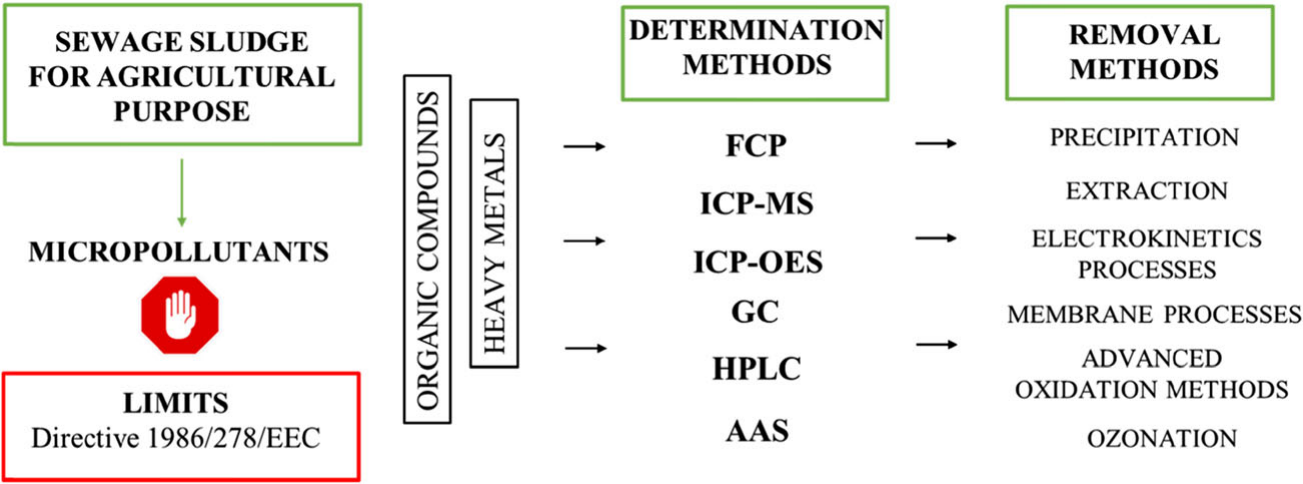
Micropollutants in sludge

Both sewage sludge and products from its chemical treatment may contain certain amounts of micropollutants (i.e. heavy metals, nanoparticles, various persistent organic compounds, microplastics (Zhang and Chen 2020)) that need to be removed to fully utilize the material for various purposes (Fig. 4). Directive 1986/278/EEC prescribes the quality of sewage sludge that can be used in agriculture but without specifying techniques for reducing micropollutants limits. This directive has been found to be outdated and is currently being improved (Fijalkowski et al. 2017). The introduction of micropollutants into the soil can disrupt microbiological processes, affect crop quality and enter the food chain; hence, their concentrations need to be constantly monitored. A positive signal is the decreasing concentration of heavy metals in sludge in developed countries (i.e. Sweden) where effective environmental protection has been successfully implemented (Kirchmann et al. 2017).

Various methods can remove organic micropollutants. Among them, oxidation seems to be the most effective, e.g. ozonation, the advanced oxidation methods (AOP) and the assistance of ultrasounds or thermal processes (Semblante et al. 2015). Heavy metals can be removed by extraction, electrokinetic processes, washing or membrane techniques.

Heavy metal ions can be removed selectively from active sludge treatment solutions by precipitation in the form of sulphides (Seaborne process) and extraction to the organic phase (PASCH process) (Blöcher et al. 2012). Surfactants and chelating agents that effectively bind heavy metal ions can be used for sludge chemical washing (Wu et al. 2015). Biosurfactants of microbiological origin and compounds of plant origin are proposed to reduce the environmental burden. Plant compounds derived from *H. acerba* removed more than 75% of Cu and more than 50% of Pb and Cd from sewage sludge (Xu et al. 2020). Sadly, this method generates large amounts of liquid waste.

Electrokinetic technology (ET) makes us of electrodes whose current attracts migrating heavy metal ions. To prevent the precipitation of the insoluble metal salts, it is necessary to keep them in a dissolved form, e.g., by using chelating compounds (Park et al. 2010) or biosurfactants. The effectiveness of heavy metal ions removal using biosurfactants (rhamnolipid, saponin and sophorolipid) resulted in the elimination of 55–74% of heavy metals ions (Tang et al. 2018). There are also known processes combining electrokinetic treatment and bioleaching (Xu et al. 2017). Although the method is simple, it can be used under various conditions (in situ and ex situ), is relatively inexpensive and environmentally friendly, but has not yet found an application on a larger than laboratory scale (Ma et al. 2020).

Membrane methods, which are widely used in industrial applications, have a high potential for the removal of heavy metal ions. Nanofiltration, a pressure-driven membrane process that uses nanoporous membranes with extra charges in their pores, favouring Donnan’s exclusion, is excellent for separating polyvalent ions, including heavy metals. This process was applied for the removal of heavy metal ions during dewatering of sludge (Hedayatipour et al. 2017). Appropriate selection of the membrane and the pH of the solution will also enable selective separation of valuable nutrients, such as phosphorus or nitrogen compounds (Schütte et al. 2015; Thong et al. 2016). Sometimes, a two-stage membrane system can be used. The first step in ultrafiltration separates the macromolecular components and the permeate is then moved to the nanofiltration separation, where separation of ions takes place. Under reduced pH conditions, phosphorus compounds are in the form of phosphoric acid and H_2_PO_4_^−^ ions, so they are easily separated from heavy metal ions retained on the membrane (Blöcher et al. 2012). Electrodialysis (ED) is an electric membrane technique in which the cooperation of ion-selective membranes in an electric field results in the selective transport of positively and negatively charged ions to appropriate compartments. This process facilitates selective separation of heavy metal ions from other valuable components, including phosphorus (Ebbers et al. 2015). Supported liquid membranes (SLM) can also be used to remove metal ions from leaching solutions. *The pore-immobilized membrane carrier dissolved in organic solvent enables selective transport of heavy metal ions from the sludge leaching solutions* (Yesil and Tugtas 2019). Despite the high selectivity of the process, diffusion seems to be the limiting stage, significantly reducing transport efficiency. Donnan dialysis (DD) is an effective method for the selective separation of heavy metal ions from hydrolysates. The solution obtained by membrane separation meets the requirements for heavy metal limits in fertilizers (Uysal et al. 2017). Undoubtedly, the need to remove unwanted components from sludge chemical treatment solutions is a crucial task. Given the efficiency and selectivity of the separation and the possibility of larger-scale applications, membrane methods have great potential.

## Agricultural use of sewage sludge

**Fig. 5:**
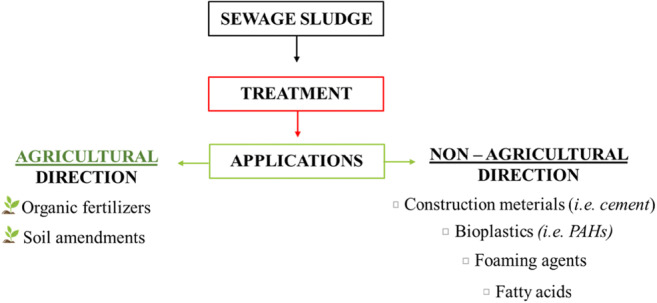
Directions of sewage sludge using

Due to the availability of nutrients and high organic matter content, sewage sludge may prove to be useful in low-soil remediation (Fig. 5). Such organic waste can be used as organic fertilizer or biopreparation that improves soil conditions for plant cultivation, as it positively affects soil fertility or water retention (Pérez-Gimeno et al. 2019). Removal of toxic metals and pathogens from sewage sludge by composting allows their use as soil amendments. Phung et al. (2020) demonstrated the usefulness of this waste as a low-cost alternative to rice cultivation. In addition, treated wastewater was used for irrigation. Pot tests were carried out in the greenhouse. Studies have shown that yields with similar rice protein content (7.5%) were obtained by applying twice the dose of sewage sludge without irrigation with sewage (2.6g N/pot) compared to mineral fertilizer. Unfortunately, this practice increased the As content (0.34 mg/kg) in rice grains. However, no accumulation of toxic metals was observed with a smaller SS dose (1.3g N/pot) in combination with treated sewage. This solution brought unexpectedly favourable results: rice protein content was 25% and the yield was 27% higher (Phung et al. 2020). A 2-year sunflower study by Koutroubas et al. (2020) proved that sewage sludge positively affects soil water retention. Thanks to this, the plants grew faster, regardless of precipitation (Koutroubas et al. 2020).

Tóth and Moloi (2019) investigated the potential application of lime sewage sludge as fertilizers correcting Fe deficiency. Due to different nutritional needs, corn and sunflower were used for testing. Seedlings were grown for 12 days (sewage sludge doses: 2 and 4 g/L). Even a small dose of sewage sludge (2g/L) proved to be effective for both plant species. Compared to the controls, 30% and 22% longer roots were noted for sunflower and corn. Studies have confirmed that sewage sludge can be an alternative to currently used mineral fertilizers (Tóth and Moloi 2019). However, the use of biosolids in agriculture can lead to increased metal accumulation in soil and plants. Latare et al. (2014) examined the effect of SS fertilization on the content of toxic elements in greenhouse studies. A 45% higher rice yield was obtained after applying biosolid (40t/ha) compared to the control. The content of soil nutrients (S, P, Fe, Mn, Zn) increased due to SS fertilization. Despite the benefits, there occurred Cd accumulation in rice grains above acceptable levels (at SS dose of 20t/ha and more) (Latare et al. 2014).

Uysal et al. (2017) proposed the use of sludge from acid hydrolysis for fertilizing. The obtained struvite was purified with Donnan dialysis, which reduced toxic elements to acceptable fertilizer levels. The fertilizer was characterized by a high content of phosphorus, potassium and iron. In another case, the hydrolysis of sludge proteins in hot hydrochloric acid is an effective process, allowing the extraction of up to 78% of proteins. The released amino acids can serve as microelement chelators and, in this form, can be used as fertilizers (Liu et al. 2009). Alkaline hydrolysates from fermented biowaste can be applied as plant growth biostimulants or bio-regulators. Rapeseed studies have shown that hydrolysates used as fungicides against *Leptosphaeria maculans* reduced necrosis (by 42–56% and 31–37% for foliar and soil application, respectively) and biomass increase (by 9% compared to control) (Jindřichová et al. 2018).

## Non-agricultural directions

There are a few reports of non-agricultural use of sewage sludge in the literature. They mainly concern the manufacture of construction materials, production of bioplastics and surfactants. Zhou et al. (2020) presented the possibility of using sewage sludge for the recovery of regenerative components and energy production. The method based on a combination of anaerobic fermentation and alkalization promotes the reduction of acetogenesis in the microbial environment, which affects the production of short-chain fatty acids, particularly acetic acid, which is an intermediate product for energy production.

Incineration of sewage sludge eliminates waste toxicity and odour. Sewage sludge ashes as a product of this process are characterized by lower mass and volume, which makes them easier to manage. The possibility of reusing SSA depends mainly on their chemical composition. Due to the presence of Al, Ca, Si and Fe and pozzolanic properties, SSA can be applied in concrete production as a filler (replacement for fine aggregate) or as cement material. Vouk et al. (2017) prepared mortars in which cement was replaced by SSA (10–20% fraction). They used ashes without lime and ashes where lime was added during stabilization or combustion of sewage sludge. The more SSA was added, the more porous the samples were. It was noted that the presence of lime increased the porosity of the material. The best mechanical properties (98% compressive strength, 95% bending strength) had a cement mixture with 10% SSA (without lime, sludge burning at 800 °C). Increasing the combustion temperature of sewage sludge is not recommended (up to 1000 °C) because the material’s mechanical properties deteriorate. Sludge conditioning with lime is also unnecessary because it does not significantly affect the quality of the material and causes nitrogen losses (Vouk et al. 2017).

Plastic pollution is a global problem. Microorganisms can be used to produce biodegradable materials such as polyhydroxyalkanoates (PAHs). Sewage sludge as a mixture of bacterial culture could be a potential raw material. This approach could reduce the problem of sludge generated from treatment plants, minimize environmental damage (less plastic waste) and lower production costs. Yan et al. (2008) successfully produced PAH applying various active sludge doses (5–20g/L). A medium in the form of acetate (5–20 mg/L) was added to bioreactors, and the process was carried out at 25 °C (pH 7) under optimal conditions (SS and acetone concentration: 15g/L and 10g/L, respectively), yielding 39.6% of bioplastic, which was finally separated by extraction (Yan et al. 2008).

Hydrolyzed proteins from sludge are also used as foaming agents, particularly when the main product is polypeptides (Gao et al. 2020a). The presence of hydrolyzed proteins showing foaming properties were also demonstrated in the filtrate after the disintegration of sludge from the brewing industry. In this way, complex protein foaming agent (CPFA) was obtained to produce cellular concrete (Li et al. 2017). In turn, microorganisms, especially bacteria, in pretreatment lead to the formation of surfactants (Jacques et al. 2005). García Becerra et al. (2010) extracted biosolids from sewage sludge to produce surfactants in an alkaline environment.

## Future perspectives

Excess sludge from biological wastewater treatment plants consists of bacteria, protozoa, algae, metazoans, organic matter and an organic matrix containing proteins, nucleic acids and polysaccharides. The protein content is 30–60% (Gao et al. 2020a, b). Proteins isolated from sewage sludge can find diversified applications and can be used as animal feed, foaming agents and liquid fertilizers (Qin et al. 2018). In light of the global feed protein deficit and the demand for fertilizing amino acids, the valorization of sewage sludge as a source of protein seems to be a promising and needed direction.

Different sewage sludge valorizations that have the potential for future applications have been developed: chemical, biochemical and physical protein extraction and hybrid methods. These methods differ in extraction efficiency and the degree of protein hydrolysis (Gao et al. 2020a, b). An interesting direction may be thermal-alkaline conditioning, where alkali cause solubilization of biological matter and further disintegrates activated sludge flocs (Huang et al. 2019).

The key in future implementation of sewage sludge valorization technologies is to identify process parameters and their impact on the rate of protein extraction, energy consumption, material consumption and cost, quality of protein hydrolyzate and efficiency of sludge dewatering (Xiao & Zhou 2020; Gao et al. 2020a, b).

The perspectives of implementation of sewage sludge management directions include anaerobic digestion, co-digestion, incineration with energy recovery, co-incineration in combined heat and power plants or with other waste, in residential and road construction, pyrolysis, gasification, hydrolysis at high temperature, landfilling, transformation to fertilizer for agricultural and non-agricultural use. Incineration or co-incineration with energy recovery and soil application is commonly used (Liu et al. 2009).

Processed sludge as fertilizer or soil improvement material offers promising applications (Collivignarelli et al. 2015). However, compared to mineral fertilizers, the content of fertilizer components is low. This makes it necessary to use a high tonnage of the material per hectare. The key determinant of agricultural use is the ratio of the fertilizer to toxic elements and persistent organic pollutants (POPs) (Liu et al. 2009).

Due to the implementation of circular economy principles, increasing fees for sewage sludge utilization and growing fertilizer prices, interest in agricultural and non-agricultural use of sewage sludge has risen in recent years (Xiao & Zhou 2020). Sewage sludges contain significant levels of major nutrients (NPK) and secondary nutrients (CaMgS). The sludges also contain micronutrients: Fe, Mn and Co. They are rich in organic matter (approx. 40% m/m). The content of toxic elements regulated in the fertilizer (As, Cd, Pb, Hg) is relatively low. The problem of managing sewage sludge for commercial purposes is the presence of *coli* bacteria. Therefore, excess sewage sludge requires processing and sanitization (Boumalek et al. 2019).

The development of sewage valorization technologies is limited by legal regulations, in which some European countries do not allow the use of stabilized sewage sludge in agriculture. It is worth mentioning that stabilization is a biological process. On the other hand, the law imposes restrictions on sludge disposal in landfills, which applies to sludge containing > 5% organic matter. The practical solution seems to be the combustion of sludge (Boehler & Siegrist 2006). An interesting application is the disintegration of sewage sludge, which causes the cells to break down, releasing their contents, which significantly facilitates its biological utilization in aerobic and anaerobic conditions. This reduces the amount of excess sludge (Boehler & Siegrist 2006).

## Conclusions

The global amount of sewage sludge has increased significantly in recent years. Therefore, it is necessary to quickly develop and implement different sludge valorization technologies in industrial practice, taking into account the favourable high protein content. Future development directions can include feed additives, foliar fertilizers and flame retardants.

The purpose of this work was to review practical methods of the valorization of excess sewage sludge. We underscored the need for a methodological approach to regulatory planning in agricultural and non-agricultural use of sewage sludge. Approximately half of the sludge is used for agricultural purposes. Various need to be addressed yet. Sewage sludge should be processed in order to improve safety and to achieve the effect of sanitation and increase the bioavailability of fertilizer components to plants. The problems that may arise are the presence of toxic elements, the variable quality of sewage sludge (depending on the treatment plant or even the season of the year), and odour emission. It is necessary to define persistent organic pollutants controlled by law both at the local level and regulated by European directives.

The discussion of development directions focused on effective processes for the recovery of substances from biological waste, among others phosphorus, mineral compounds (e.g., Ca, Mg) with a preferential place of development on-site of their generation. We have pointed out the substances that can be recovered, considering the economic and practical aspects. The determination of both elemental (potential content of heavy metals) and material (e.g. the content of proteins) composition to identify useful substances that interfere with or limit a given application is key to indicating the directions of waste management of biological origin. When selecting agricultural and non-agricultural uses of bio-waste, soil-forming and fertilizing values ​​were taken into account. Bio-wastes are usually a source of elements—such as N, P, K, Ca, Mg, Cu and Mn—that are essential in the cultivation of plants. Their soil application has a beneficial effect on soil organic matter content, sorption capacity and an overall improvement in physical properties. Sanitary and epidemiological safety is essential, which is why bio-wastes require processing according to selected technologies that aim to improve the bioavailability of nutrients and reduce hazards before entering the soil. We have paid particular attention to preventing secondary pollution. The use of bio-waste is a solution dedicated to the circular economy. Agricultural and non-agricultural biowaste farming is a synergistic action with resource recovery.

## Abbreviations

AD: Anaerobically digested
AKT: Alkaline-thermal
AOP: Advanced oxidation methods
AT: Acid-thermal
CaMgS: Secondary nutrients
CD: Charge density
CE: Composite enzyme
COD: Chemical oxygen demand
CPFA: Complex protein foaming agent
DD: Donnan dialysis
d.m: Dry matter
ED: Electrodialysis
EDTA: Ethylenediaminetetraacetic acid
ET: Electrokinetic technology
EU: European Union
MW: Molecular weight
NPK: Major nutrients
PAH: Polyhydroxyalkanoates
PASCH: Extraction process to the organic phase
POP: Persistent organic pollutants
SE: Single enzyme
SLM: Supported liquid membranes
SS: Sewage sludge
SSA: Sewage sludge ash
TE: Thermal assisted enzyme
TPT: Thermal pretreatment
USE: Ultrasonic assisted enzyme
WWTP: Wastewater treatment plant
XRF: X-ray fluorescence

## References

[CR1] Ahmad T, Ahmad K, Alam M (2016). Sustainable management of water treatment sludge through 3’R’ concept. J Clean Prod.

[CR2] Anjum M, Al-Makishah NH, Barakat MA (2016). Wastewater sludge stabilization using pretreatment methods. Process Saf Environ Prot.

[CR3] Barber WPF (2016). Thermal hydrolysis for sewage treatment: a critical review. Water Res.

[CR4] Beszédes S, Kertész S, László Z, Szabó G, Hodúr C, (2009) Biogas Production of Ozone and/or Microwave-Pretreated Canned Maize Production Sludge. Ozone: Science & Engineering 31 (3):257–261. 10.1080/01919510902841218

[CR5] Bi W, Li Y, Hu Y (2014). Recovery of phosphorus and nitrogen from alkaline hydrolysis supernatant of excess sludge by magnesium ammonium phosphate. Bioresour Technol.

[CR6] Bian B, Zhang L, Zhang Q, Zhang S, Yang Z, Yang W (2018). Coupled heating/acidification pretreatment of chemical sludge for dewatering by using waste sulfuric acid at low temperature. Chemosphere.

[CR7] Blöcher C, Niewersch C, Melin T (2012). Phosphorus recovery from sewage sludge with a hybrid process of low pressure wet oxidation and nanofiltration. Water Res.

[CR8] Boehler M, Siegrist H (2006) Potential of activated sludge disintegration. Water Sci Technol 53(12):207–216. 10.2166/wst.2006.423 16889257

[CR9] Boumalek W, Kettab A, Bensacia N, Bruzzoniti MC, Othman DB, Mandi L, Chabaca MN, Benziada S (2019). Specification of sewage sludge arising from a domestic wastewater treatment plant for agricultural uses. Desalin Water Treat..

[CR10] Cai M, Hu J, Lian G, Xiao R, Song Z, Jin M, Dong C, Wang Q, Luo D, Wei Z (2018). Synergetic pretreatment of waste activated sludge by hydrodynamic cavitation combined with Fenton reaction for enhanced dewatering. Ultrason Sonochem.

[CR11] Carrère H, Dumas C, Battimelli A, Batstone DJ, Delgenès JP, Steyer JP, Ferrer I (2010). Pretreatment methods to improve sludge anaerobic degradability: a review. J Hazard Mater.

[CR12] Cassini ST, Andrade MCE, Abreu TA, Keller R, Gonçalves RF (2006). Alkaline and acid hydrolytic processes in aerobic and anaerobic sludges: effect on total EPS and fractions. Water Sci Technol.

[CR13] Cecen F, Gursoy G (2000). Characterization of landfill leachates and studies on heavy metal removal. J Environ Monit.

[CR14] Chen Y, Jiang S, Yuan H, Zhou Q, Gu G (2007). Hydrolysis and acidification of waste activated sludge at different pHs. Water Res.

[CR15] Chen N, Tao S, Xiao K, Liang S, Yang J, Zhang L (2020). A one-step acidification strategy for sewage sludge dewatering with oxalic acid. Chemosphere.

[CR16] Cieślik B, Konieczka P (2017) A review of phosphorus recovery methods at various steps of wastewater treatment and sewage sludge management. The concept of “no solid waste generation” and analytical methods. J Clean Prod. 10.1016/j.jclepro.2016.11.116

[CR17] Cies̈lik BM, Namies̈nik J, Konieczka P (2015). Review of sewage sludge management: Standards, regulations and analytical methods. J Clean Prod.

[CR18] Collivignarelli MC, Abbà A, Padovani S, Frascarolo M, Sciunnach D, Turconi M, Orlando M (2015) Recovery of sewage sludge on agricultural land in Lombardy: Current issues and regulatory scenarios. Environ Eng Manag J 14:1477–1486. 10.30638/eemj.2015.159

[CR19] Ebbers B, Ottosen LM, Jensen PE (2015). Electrodialytic treatment of municipal wastewater and sludge for the removal of heavy metals and recovery of phosphorus. Electrochim Acta.

[CR20] Eurostat - Data Explorer [WWW Document], 2020. URL https://appsso.eurostat.ec.europa.eu/nui/show.do?dataset=envwwspd&lang=en (Accessed 6.23.20).

[CR21] Fang W, Zhang X, Zhang P, Wan J, Guo H, Ghasimi DSM, Morera XC, Zhang T (2020). Overview of key operation factors and strategies for improving fermentative volatile fatty acid production and product regulation from sewage sludge. J Environ Sci (China).

[CR22] Farooq R, Farooq R, Rehman F, Baig S, Sadique M, Khan S, Farooq U (2009). The effect of ultrasonic irradiation on the anaerobic digestion of activated sludge. World Appl Sci J.

[CR23] Feki E, Khoufi S, Loukil S, Sayadi S (2015). Improvement of anaerobic digestion of waste-activated sludge by using H2O2 oxidation, electrolysis, electro-oxidation and thermo-alkaline pretreatments. Environ Sci Pollut Res.

[CR24] Fijalkowski K, Rorat A, Grobelak A, Kacprzak MJ (2017). The presence of contaminations in sewage sludge – the current situation. J Environ Manag.

[CR25] Gao J, Weng W, Yan Y, Wang Y, Wang Q (2020). Comparison of protein extraction methods from excess activated sludge. Chemosphere.

[CR26] Gao N, Kamran K, Quan C, Williams PT (2020). Thermochemical conversion of sewage sludge: a critical review. Prog Energy Combust Sci.

[CR27] García Becerra FY, Acosta EJ, Grant Allen D (2010). Alkaline extraction of wastewater activated sludge biosolids. Bioresour Technol.

[CR28] Gray SR, Ritchie CB (2006). Effect of organic polyelectrolyte characteristics on floc strength. Colloids Surfaces A Physicochem. Eng. Asp..

[CR29] Grübel K, Kuglarz M, Wacławek S, Padil VVT, Černík M, Varma RS (2019). Microwave-assisted sustainable co-digestion of sewage sludge and rapeseed cakes. Energy Convers Manag.

[CR30] Güney K, Weidelener A, Krampe J (2008). Phosphorus recovery from digested sewage sludge as MAP by the help of metal ion separation. Water Res.

[CR31] Healy MG, Fenton O, Forrestal PJ, Danaher M, Brennan RB, Morrison L (2016). Metal concentrations in lime stabilised, thermally dried and anaerobically digested sewage sludges. Waste Manag.

[CR32] Hedayatipour M, Jaafarzadeh N, Ahmadmoazzam M (2017). Removal optimization of heavy metals from effluent of sludge dewatering process in oil and gas well drilling by nanofiltration. J Environ Manag.

[CR33] Huang Y, Wang Y, Liu S, Huang W, He L, Zhou J (2019) Enhanced hydrolysis-acidification of high-solids and low-organic-content sludge by biological thermal-alkaline synergism. Bioresour Technol 294:122234. 10.1016/j.biortech.2019.122234 10.1016/j.biortech.2019.122234 31610488

[CR34] Inglezakis VJ, Zorpas AA, Karagiannidis A, Samaras P, Voukkali I, Sklari S (2011). European Union legislation on sewage sludge management. Fresenius Environmental Bulletin..

[CR35] Jacques RJS, Santos EC, Bento FM, Peralba MCR, Selbach PA, Sá ELS, Camargo FAO (2005). Anthracene biodegradation by Pseudomonas sp. isolated from a petrochemical sludge landfarming site. Int Biodeterior Biodegrad.

[CR36] Janas M, Zawadzka A, Cichowicz R (2018). The influence of selected factors on leaching of metals from sewage sludge. Environ Sci Pollut Res.

[CR37] Jindřichová B, Burketová L, Montoneri E, Francavilla M (2018). Biowaste-derived hydrolysates as plant disease suppressants for oilseed rape. J Clean Prod.

[CR38] Khanal SK, Grewell D, Sung S, van Leeuwen J(H) (2007). Ultrasound applications in wastewater sludge pretreatment: a review. Crit Rev Environ Sci Technol.

[CR39] Kim J, Park C, Kim T-H, Lee M, Kim S, Kim S-W, Lee J (2003). Effects of various pretreatments for enhanced anaerobic digestion with waste activated sludge. J Biosci Bioeng.

[CR40] Kim M, Han DW, Kim DJ (2015). Selective release of phosphorus and nitrogen from waste activated sludge with combined thermal and alkali treatment. Bioresour Technol.

[CR41] Kirchmann H, Börjesson G, Kätterer T, Cohen Y (2017). From agricultural use of sewage sludge to nutrient extraction: a soil science outlook. Ambio.

[CR42] Koutroubas SD, Antoniadis V, Damalas CA, Fotiadis S (2020). Sunflower growth and yield response to sewage sludge application under contrasting water availability conditions. Ind Crop Prod.

[CR43] Latare AM, Kumar O, Singh SK, Gupta A (2014). Direct and residual effect of sewage sludge on yield, heavy metals content and soil fertility under rice-wheat system. Ecol Eng.

[CR44] Lee CH, Liu JC (2000). Enhanced sludge dewatering by dual polyelectrolytes conditioning. Water Res.

[CR45] Li H, Jin Y, Mahar RB, Wang Z, Nie Y (2008). Effects and model of alkaline waste activated sludge treatment. Bioresour Technol.

[CR46] Li H, Li C, Liu W, Zou S (2012). Optimized alkaline pretreatment of sludge before anaerobic digestion. Bioresour Technol.

[CR47] Li H, Zou S, Li C, Jin Y (2013). Alkaline post-treatment for improved sludge anaerobic digestion. Bioresour Technol.

[CR48] Li P, Deng F, Zhu H, Guan S, Huang S (2017). Study of a complex protein foaming agent from disintegrated brewery sludge supernatant. Desalin Water Treat.

[CR49] Liu Y, Kong S, Li Y, Zeng H (2009). Novel technology for sewage sludge utilization: Preparation of amino acids chelated trace elements (AACTE) fertilizer. J Hazard Mater.

[CR50] Lopes BC, Machado EC, Rodrigues HF, Leal CD, de Araújo JC, Teixeira de Matos A (2020). Effect of alkaline treatment on pathogens, bacterial community and antibiotic resistance genes in different sewage sludges for potential agriculture use. Environ Technol.

[CR51] Ma D, Su M, Qian J, Wang Q, Meng F, Ge X, Ye Y, Song C (2020). Heavy metal removal from sewage sludge under citric acid and electroosmotic leaching processes. Sep Purif Technol.

[CR52] Madrid F, Rubio-Bellido M, Morillo E (2020). Extraction of nonylphenol, pyrene and phenanthrene from sewage sludge and composted biosolids by cyclodextrins and rhamnolipids. Sci Total Environ.

[CR53] McSwain BS, Irvine RL, Hausner M, Wilderer PA (2005). Composition and distribution of extracellular polymeric substances in aerobic flocs and granular sludge. Appl Environ Microbiol.

[CR54] Neyens E, Baeyens J, Weemaes M, De Heyder B (2003). Hot acid hydrolysis as a potential treatment of thickened sewage sludge. J Hazard Mater.

[CR55] Neyens E, Baeyens J, Dewil R, De Heyder B (2004). Advanced sludge treatment affects extracellular polymeric substances to improve activated sludge dewatering. J Hazard Mater.

[CR56] Park S-Y, Park G-Y, Kim D-H, Yang J-S, Baek K (2010). Electrokinetic separation of heavy metals from wastewater treatment sludge. Sep Sci Technol.

[CR57] Pastor L, Marti N, Bouzas A, Seco A (2008). Sewage sludge management for phosphorus recovery as struvite in EBPR wastewater treatment plants. Bioresour Technol.

[CR58] Paz-Ferreiro J, Nieto A, Méndez A, Askeland M, Gascó G (2018). Biochar from biosolids pyrolysis: a review. Int J Environ Res Public Health.

[CR59] Pérez-Gimeno A, Navarro-Pedreño J, Almendro-Candel MB, Gómez I, Zorpas AA (2019). The use of wastes (organic and inorganic) in land restoration in relation to their characteristics and cost. Waste Manag Res.

[CR60] Phung LD, Ichikawa M, Pham DV, Sasaki A, Watanabe T (2020). High yield of protein-rich forage rice achieved by soil amendment with composted sewage sludge and topdressing with treated wastewater. Sci Rep.

[CR61] Pilli S, Yan S, Tyagi RD, Surampalli RY (2015). Thermal pretreatment of sewage sludge to enhance anaerobic digestion: a review. Crit Rev Environ Sci Technol.

[CR62] Pilnáček V, Innemanová P, Šereš M, Michalíková K, Stránská, Wimmerová L, Cajthaml T (2019). Micropollutant biodegradation and the hygienization potential of biodrying as a pretreatment method prior to the application of sewage sludge in agriculture. Ecol Eng.

[CR63] Qin L, Yan YX, Gao JL, Miao CY (2018) Extraction of protein from excess sludge by enzymatic hydrolysis. IOP Conf. Ser. Earth Environ. Sci. 191. 10.1088/1755-1315/191/1/012069

[CR64] Santos AF, Santos CP, Matos AM, Cardoso O, Quina MJ (2020). Effect of thermal drying and chemical treatments with wastes on microbiological contamination indicators in sewage sludge. Microorganisms.

[CR65] Sapkaite I, Barrado E, Fdz-Polanco F, Pérez-Elvira SI (2017). Optimization of a thermal hydrolysis process for sludge pretreatment. J Environ Manag.

[CR66] Saveyn H, Meersseman S, Thas O, Van Der Meeren P (2005). Influence of polyelectrolyte characteristics on pressure-driven activated sludge dewatering. Colloids Surfaces A Physicochem. Eng. Asp..

[CR67] Schütte T, Niewersch C, Wintgens T, Yüce S (2015). Phosphorus recovery from sewage sludge by nanofiltration in diafiltration mode. J Membr Sci.

[CR68] Semblante GU, Hai FI, Huang X, Ball AS, Price WE, Nghiem LD (2015). Trace organic contaminants in biosolids: impact of conventional wastewater and sludge processing technologies and emerging alternatives. J Hazard Mater.

[CR69] Siami S, Aminzadeh B, Karimi R, Hallaji SM (2020). Process optimization and effect of thermal, alkaline, H2O2 oxidation and combination pretreatment of sewage sludge on solubilization and anaerobic digestion. BMC Biotechnol.

[CR70] Stylianou MA, Kollia D, Haralambous KJ, Inglezakis VJ, Moustakas KG, Loizidou MD (2007). Effect of acid treatment on the removal of heavy metals from sewage sludge. Desalination.

[CR71] Suárez-Iglesias O, Urrea JL, Oulego P, Collado S, Díaz M (2017). Valuable compounds from sewage sludge by thermal hydrolysis and wet oxidation. A review. Sci Total Environ.

[CR72] Taboada-Santos A, Braz GHR, Fernandez-Gonzalez N, Carballa M, Lema JM (2019). Thermal hydrolysis of sewage sludge partially removes organic micropollutants but does not enhance their anaerobic biotransformation. Sci Total Environ.

[CR73] Taboada-Santos A, Lema JM, Carballa M (2019). Energetic and economic assessment of sludge thermal hydrolysis in novel wastewater treatment plant configurations. Waste Manag.

[CR74] Tang J, He J, Xin X, Hu H, Liu T (2018). Biosurfactants enhanced heavy metals removal from sludge in the electrokinetic treatment. Chem Eng J.

[CR75] Thapa KB, Qi Y, Hoadley AFA (2009). Interaction of polyelectrolyte with digested sewage sludge and lignite in sludge dewatering. Colloids Surf A Physicochem Eng Asp.

[CR76] Thong Z, Cui Y, Ong YK, Chung TS (2016). Molecular design of nanofiltration membranes for the recovery of phosphorus from sewage sludge. ACS Sustain Chem Eng.

[CR77] Tian X, Wang C, Trzcinski AP, Lin L, Ng WJ (2015). Insights on the solubilization products after combined alkaline and ultrasonic pretreatment of sewage sludge. J Environ Sci (China).

[CR78] Tóth B, Moloi MJ (2019). The use of industrial waste materials for alleviation of iron deficiency in sunflower and maize. Int J Recycl Org Waste Agric.

[CR79] Tyagi VK, Lo SL (2013). Sludge: a waste or renewable source for energy and resources recovery?. Renew Sust Energ Rev.

[CR80] Tyagi VK, Lo SL (2013). Microwave irradiation: a sustainable way for sludge treatment and resource recovery. Renew Sust Energ Rev.

[CR81] Uysal, A., Tuncer, D., Kir, E., Koseoglu, T.S., 2016. Phosphorus recovery from hydrolysed sewage sludge liquid containing metals using Donnan dialysis, in: Proceedings of the 2nd World Congress on New Technologies (NewTech’16). 10.11159/icepr16.125

[CR82] Uysal A, Tuncer D, Kir E, Koseoglu TS (2017). Recovery of nutrients from digested sludge as struvite with a combination process of acid hydrolysis and Donnan dialysis. Water Sci Technol.

[CR83] Vigueras-Carmona SE, Ramírez F, Noyola A, Monroy O (2011). Effect of thermal alkaline pretreatment on the anaerobic digestion of wasted activated sludge. Water Sci Technol.

[CR84] Vlyssides AG, Karlis PK (2004). Thermal-alkaline solubilization of waste activated sludge as a pretreatment stage for anaerobic digestion. Bioresour Technol.

[CR85] Vouk D, Nakic D, Štirmer N, Baricevic A (2017). Effect of lime addition during sewage sludge treatment on characteristics of resulting SSA when it is used in cementitious materials. Water Sci Technol.

[CR86] Wang Y, Zhang T, Westerhoff P, Jiang R, Ren H, Yang Y, Li Z (2017). Microwave-assisted digestion and NaOH treatment of waste-activated sludge to recover phosphorus by crystallizing struvite. Environ Technol (United Kingdom).

[CR87] Wong JWC, Fang M (2000). Effects of lime addition on sewage sludge composting process. Water Res.

[CR88] Wu Q, Cui Y, Li Q, Sun J (2015). Effective removal of heavy metals from industrial sludge with the aid of a biodegradable chelating ligand GLDA. J Hazard Mater.

[CR89] Xiao K, Zhou Y (2020) Protein recovery from sludge: A review. J Clean Prod 249:119373. 10.1016/j.jclepro.2019.119373

[CR90] Xu L, Geelen D (2018) Developing biostimulants from agro-food and industrial by-products. Front Plant Sci 9. 10.3389/fpls.2018.01567 10.3389/fpls.2018.01567 PMC6218572 30425724

[CR91] Xu Y, Zhang C, Zhao M, Rong H, Zhang K, Chen Q (2017). Comparison of bioleaching and electrokinetic remediation processes for removal of heavy metals from wastewater treatment sludge. Chemosphere..

[CR92] Xu ZX, Song H, Deng XQ, Zhang YY, Xue-Qin M, Tong SQ, He ZX, Wang Q, Shao YW, Hu X (2019). Dewatering of sewage sludge via thermal hydrolysis with ammonia-treated Fenton iron sludge as skeleton material. J Hazard Mater.

[CR93] Xu X, Yang Y, Wang G, Zhang S, Cheng Z, Li T, Yang Z, Xian J, Yang Y, Zhou W (2020). Removal of heavy metals from industrial sludge with new plant–based washing agents. Chemosphere.

[CR94] Yan S, Subramanian SB, Tyagi RD, Surampalli RY (2008). Bioplastics from waste activated sludge-batch process. Pract. Period. Hazardous, Toxic, Radioact. Waste Manag.

[CR95] Yesil H, Tugtas AE (2019). Removal of heavy metals from leaching effluents of sewage sludge via supported liquid membranes. Sci Total Environ.

[CR96] Zhang Z, Chen Y (2020). Effects of microplastics on wastewater and sewage sludge treatment and their removal: a review. Chem Eng J.

[CR97] Zhang P, Zhang G, Wang W (2007). Ultrasonic treatment of biological sludge: floc disintegration, cell lysis and inactivation. Bioresour Technol.

[CR98] Zhang H, Gao Z, Liu Y, Ran C, Mao X, Kang Q, Ao W, Fu J, Li J, Liu G, Dai J (2018). Microwave-assisted pyrolysis of textile dyeing sludge, and migration and distribution of heavy metals. J Hazard Mater.

[CR99] Zhen G, Lu X, Kato H, Zhao Y, Li YY (2017). Overview of pretreatment strategies for enhancing sewage sludge disintegration and subsequent anaerobic digestion: current advances, full-scale application and future perspectives. Renew Sust Energ Rev.

[CR100] Zheng NY, Lee M, Lin YL (2020). Co-processing textile sludge and lignocellulose biowaste for biofuel production through microwave-assisted wet torrefaction. J Clean Prod.

[CR101] Zhou J, Parker W, Laha S (2007). Biosolids and sludge management. Water Environ Res.

[CR102] Zhou L, Gao Y, Yu K, Zhou H, De Costa YG, Yi S, Zhuang WQ (2020). Microbial community in in-situ waste sludge anaerobic digestion with alkalization for enhancement of nutrient recovery and energy generation. Bioresour Technol.

[CR103] Zhuravlev PV, Aleshnya VV, Marchenko BI (2019). Determination of the disinfectant action of caustic lime on the microflora of sludge of wastewater of cleaning facilities for sewerage and cattle-breeding complexes. Gig Sanit.

